# Microbial Population Differentials between Mucosal and Submucosal Intestinal Tissues in Advanced Crohn's Disease of the Ileum

**DOI:** 10.1371/journal.pone.0134382

**Published:** 2015-07-29

**Authors:** Rodrick J. Chiodini, Scot E. Dowd, William M. Chamberlin, Susan Galandiuk, Brian Davis, Angela Glassing

**Affiliations:** 1 St. Vincent Healthcare, Sisters of Charity of Leavenworth Health System, Billings, Montana, United States of America; 2 Department of Biological and Physical Sciences, Montana State University-Billings, Billings, Montana, United States of America; 3 Mr. DNA Molecular Research Laboratory, Shallowater, Texas, United States of America; 4 Department of Surgery, University of Louisville, Louisville, Kentucky, United States of America; 5 Department of Surgery, Paul L. Foster School of Medicine, Texas Tech University Health Sciences Center, El Paso, Texas, United States of America; Wageningen University, NETHERLANDS

## Abstract

Since Crohn's disease is a transmural disease, we hypothesized that examination of deep submucosal tissues directly involved in the inflammatory disease process may provide unique insights into bacterial populations transgressing intestinal barriers and bacterial populations more representative of the causes and agents of the disease. We performed deep 16s microbiota sequencing on isolated ilea mucosal and submucosal tissues on 20 patients with Crohn's disease and 15 non-inflammatory bowel disease controls with a depth of coverage averaging 81,500 sequences in each of the 70 DNA samples yielding an overall resolution down to 0.0001% of the bacterial population. Of the 4,802,328 total sequences generated, 98.9% or 4,749,183 sequences aligned with the Kingdom Bacteria that clustered into 8545 unique sequences with <3% divergence or operational taxonomic units enabling the identification of 401 genera and 698 tentative bacterial species. There were significant differences in all taxonomic levels between the submucosal microbiota in Crohn's disease compared to controls, including organisms of the Order *Desulfovibrionales* that were present within the submucosal tissues of most Crohn's disease patients but absent in the control group. A variety of organisms of the Phylum *Firmicutes* were increased in the subjacent submucosa as compared to the parallel mucosal tissue including *Ruminococcus* spp., *Oscillospira* spp., *Pseudobutyrivibrio* spp., and *Tumebacillus* spp. In addition, *Propionibacterium* spp. and *Cloacibacterium* spp. were increased as well as large increases in *Proteobacteria* including *Parasutterella* spp. and *Methylobacterium* spp. This is the first study to examine the microbial populations within submucosal tissues of patients with Crohn's disease and to compare microbial communities found deep within the submucosal tissues with those present on mucosal surfaces. Our data demonstrate the existence of a distinct submucosal microbiome and ecosystem that is not well reflected in the mucosa and/or downstream fecal material.

## Introduction

Crohn’s disease remains one of the major challenges in gastroenterology. Although the etiology remains unknown, it is now generally accepted that the Crohn's disease syndrome results, in part or in whole, from an abnormal or dysregulated immune response to specific and/or commensal bacteria arising from the intestinal lumen [1]. Supporting this suggestion is increasing evidence from genome wide association studies (GWAS) that Crohn's disease, particularly disease involving the ileum in Caucasian patients, may be related to an immune deficiency with polymorphisms detected most notably in the NOD2/CARD15 and the autophagy-associated ATG16L1 and IRGM genes [2]. These susceptibility genes are generally associated with the innate immune system’s recognition and ability to kill intracellular bacteria.

The reduction in inflammation by diverting the fecal stream by ileostomy [3], the increased bacterial translocation across the mucosal barrier [4], and the enhanced adaptive immune response to commensal bacteria [5], provide further evidence of the critical role of luminal bacteria in the etiopathogenesis and the clinical syndrome associated with Crohn's disease. Although the role of luminal bacteria as a primary cause or a secondary effect of the disease remains unclear, there is little dispute that luminal bacterial populations have a profound effect on the clinical manifestations of the disease and patient well-being.

In Crohn's disease, there are dysbiotic changes in the intestinal microbiota in both the presence of certain microbial types (diversity) as well as changes in the prevalence of certain microbial divisions or species [6]. Studying the microbiome and the disruption of this microbial community (dysbiosis) in Crohn's disease may provide a framework and principles which could govern future therapeutics aimed at shifting the intestinal microbiota to a more beneficial bacterial profile and returning the intestinal ecosystem to homeostasis and restoring a balanced microbial community structure.

We hypothesized that since Crohn's disease is a transmural disease, affecting all layers of the intestinal tract, that the examination of deep submucosal tissues directly associated with the inflammatory and disease process may provide a unique insight into bacterial populations capable of transgressing intestinal barriers and may be more representative of the causes and agents of the disease and bacteria associated with the inflammatory process. Furthermore, it was hypothesized that these bacterial populations would be less influenced by environmental factors, such as diet or therapeutic interventions, and less likely to be affected by bowel cleaning prior to endoscopy or surgery.

Our data shows that certain bacteria are significantly increased within the submucosal tissues as compared to the mucosa suggesting invasion and colonization within the diseased submucosal tissues. Data further suggests that the submucosa represents a distinct ecosystem and biological niche and that bacteria actively invading and colonizing within the submucosal tissues are different and not accurately reflected on the mucosal surfaces.

## Materials and Methods

### Patient Populations

This study was approved by the Institutional Review Boards (IRB) of the University of Louisville, Texas Tech University Health Sciences Center, and the Institutional Review Board of Billings (St Vincent Healthcare and Montana State University-Billings). Informed consent was obtained from all patients in writing on IRB-approved consent forms prior to the collection of any specimens and/or data. Patients with Crohn’s disease of the terminal ileum and controls (representing terminal ileum from non-inflammatory bowel disease (nIBD) populations) scheduled for surgical resections were recruited from the University of Louisville, Kentucky, and Texas Tech University Health Sciences Center, El Paso, and affiliated Hospitals. Detailed clinical histories were collected pre-operatively at the time of informed consent. From each Crohn's disease patient, at least a ½ sq. inch (1.27 sq. cm.) full thickness section of diseased bowel from the center of the lesion within the terminal ileum was collected at time of surgery by an operating surgeon with extensive experience with inflammatory bowel disease. Tissue samples were immediately frozen after collection. Control tissues from nIBD patients consisted of a ½ sq. inch (1.27 sq. cm.) full thickness section of macroscopically normal terminal ileum from patients undergoing resection for a variety of reasons other than Crohn's disease.

### Tissue Processing and DNA extraction

**Fig 1:**
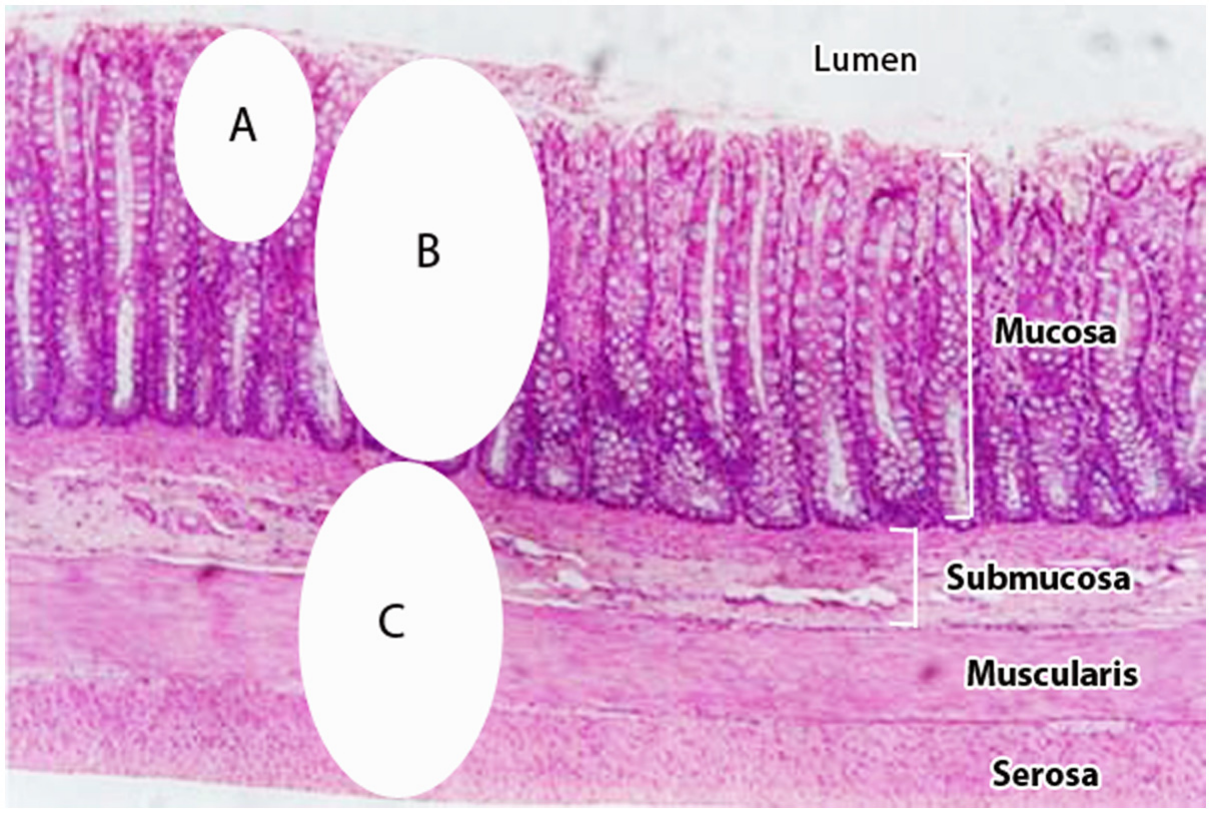
Intestinal tissues examined in present study. Mucosal tissues examined by other methods, such as snip biopsy (**A**), predominately examine microbes of the mucosal surface; Mucosal (**B**) and Submucosal (**C**) tissues examined by the present study examined bacterial populations deep within the diseased tissues.

Methods were developed to completely separate the mucosal and submucosal layers (Fig 1) of intestinal tissues and obtain representative sections beyond that accessible by simple biopsy. Tissues were processed by a modification of methods previously described [7]. An appropriate section of intestinal tissue was cut from the sample, weighed, and then vortexed in ice-cold water for 15–20 seconds at high speed to remove surface contamination. Under magnification, the mucosal layer was manually excised from the tissue and the excised mucosal layer then transferred to a microcentrifuge vial suitable for bead-beating and 1.0 ml of ice-cold 1mM DL-dithiothreitol (DTT) added and agitated for 20–30 minutes at 3000 rpm on a pulsating vortex mixer. Undissolved tissue was removed and discarded, and the supernatant centrifuged at 10,000g for 3 minutes and DNA extracted from the sediment as described below.

After physical removal of the mucosa, the remaining tissue, hereafter referred to as the submucosa, was weighed and washed in 10 ml ice-cold water by vortexing for 15–20 seconds at high speed to remove any surface adherent material. Tissues were transferred to a clean tube containing 10 ml of ice-cold DTT, vortexed for approximately 10 seconds and then placed on a rocker platform for 20–30 minutes or longer to dissolve any remaining mucus. The submucosal tissues were considered devoid of mucosa when the tissue was a uniform tan/light pink color. Once cleaned of any remaining mucosa, the submucosa was removed from the DTT and washed in 10 ml ice-cold water by vortexing for 10–20 seconds at high speed. The tissue was removed from the wash and placed into a 2ml bead-beating vial and processed as below.

DNA was extracted from the pelleted mucosal digest and solid submucosal tissue by a modification of methods employed by the Human Microbiome Project (HMP)[8] using in part the MoBio PowerMax Soil DNA Isolation Kit (MO BIO Laboratories, Inc., Carlsbad, CA). Briefly, to each vial containing tissues, 625 mg of 100 μM molecular biology grade Zirconium beads (OPS Diagnostics, Lebanon, NJ), 20 μl proteinase K (recombinant PCR grade, ~20mg/ml), 600 μl MoBio Power Bead Solution, and 60 μl MoBio Solution C1 was added. After lightly vortexing, tubes were placed in a high-energy cell disrupter (Mini-Beadbeater, Biospec Products, Inc., Bartlesville, OK) and violently agitated for 2 minutes at maximum speed and then placed in a hot water bath at 65°C for 20 minutes, followed by another bead-beating for 1 minute at maximum speed, and another incubation at 65°C for 20 minutes. The entire contents of the vials were then transferred to sterile 50 ml conical tubes and processed per manufacturer’s instructions for the MoBio PowerMax Soil DNA Isolation Kit except that the garnet beads were removed from the PowerBead tubes. DNA was concentrated in 200 μl volumes and the amount of DNA in ng per μl determined in a NanoDrop spectrophotometer at 260nm.

### 16s Metagenomic sequencing on the Illumina MiSeq platform

Amplicon sequencing using next generation technology (bTEFAP) was originally described by Dowd *et al* in 2008 [9] and has since been utilized in describing a wide range of environmental and health related microbiomes including the intestinal populations of a variety of sample types including cattle, mice, rats, humans, as well as a wide array of environmental samples [9–11]. A re-engineered modern version of bTEFAP, one of the most widely published methods for evaluating microbiota adapted to non-optical sequencing technologies on the Ion Torrent PGM and to the Illumina MiSeq platform as well as to 454 pyrosequencing, was utilized to evaluate the microbial consortiums of each sample.

The 16S rRNA gene V4 region universal Eubacterial primers 27Fmod (AGRGTTTGATCMTGGCTCAG) and 519Rmod (GTNTTACNGCGGCKGCTG) were utilized to evaluate the microbial ecology of samples on the Illumina MiSeq platform with methods based on the bTEFAP process. A single-step 30 cycle PCR using HotStarTaq Plus Master Mix Kit (Qiagen, Valencia, CA) was used under the following conditions: 94°C for 3 minutes, followed by 28 cycles of 94°C for 30 seconds; 53°C for 40 seconds and 72°C for 1 minute; after which a final elongation step at 72°C for 5 minutes was performed. After amplification, PCR products were checked in 2% agarose gel to determine the success of amplification and relative intensity of bands. Multiple samples were pooled together in equal proportions based on their molecular weight and DNA concentrations. Pooled samples were purified using calibrated Ampure XP beads. The pooled and purified PCR products were used to prepare DNA libraries according to the Illumina TruSeq DNA library preparation protocol.

Sequencing was performed at MR DNA (www.mrdnalab.com, Shallowater, TX, USA) using the Illumina MiSeq sequencing platform following the manufacturer’s guidelines. The Q25 sequence data derived from the sequencing process was processed using a standardized analysis pipeline. In brief, sequences were joined, depleted of barcodes, sequences <150bp removed, and sequences with ambiguous base calls removed. Sequences were denoised, operational taxonomic units (OTUs) generated and chimeras removed using UCHIME [12]. Operational taxonomic units were defined after removal of singleton sequences, clustering at 3% divergence (97% similarity) using UCLUST in standard default [9,13]. Final OTUs were taxonomically classified using BLASTn against a curated database derived from GreenGenes Version 13.5 (http://greengenes.lbl.gov/cgi-bin/nph-index.cgi), RDPII (http://rdp.cme.msu.edu), and NCBI (www.ncbi.nlm.nih.gov) databases (including non-bacterial sequences) and compiled into each taxonomic level by both “counts” (actual number of sequences) and “percentage” (relative proportion of sequences within each sample) files.

OTU’s were assigned taxonomic classification based on the following identity to reference sequences and taxonomic designation: >97% identity were classified at the species level; between 97% and 95% identity were designated as an unclassified species; between 95% and 90% identity were designated as an unclassified genus; between 90% and 85% identity were designated as an unclassified family; between 85% and 80% identity were designated as an unclassified order; between 80% and 77% identity were designated as an unclassified phylum; and <77% identity were designated as unclassified.

### Bacterial Quantitation

Standard bioinformatics “percentage” calculations based on the relative proportion of sequences within each sample erroneously assumes a 1:1 ratio of rRNA genes across an entire bacterial community creates bias since the actual number of rRNA genes within a bacterial population may range from 1–15 depending on the organism [14,15]. In an attempt to compensate for this bias, the actual number of sequences from the raw fasta count files was divided by the average number of rrn operons (rRNA copies) at each taxonomic level based on the Ribosomal RNA Database (rrnDB) [16]. This allowed a comparison of data based on the relative proportion of projected genomes (bacterial counts) of a particular taxonomic division, rather than the number of OTU’s aligning to a given taxonomic classification, which more accurately estimates microbial diversity and abundance [15,16].

### Human DNA Quantitation

The amount of human DNA in samples was determined using the Quantifiler Human DNA Quantification Kit (Applied Biosystems) based on the human telomerase reverse transcriptase (hTERT) gene per manufacturer’s instructions.

### Statistical Analysis

Statistical analysis was performed using a variety of computer packages including XLstat, NCSS 2007, “R” (http://www.r-project.org/) and NCSS 2010. The statistical analysis of alpha and beta diversity was performed from taxonomic classifications and phylogenetic based methods (UniFrac), not dependent on OTU assignments, as described previously using the Qiime pipeline (www.qiime.org) with standard scripts and default settings for taxa assignments (genus and higher), diversity estimates (OTUs, Chao1 index, phylogenetic distance index, Shannon index, and Simpson index), and phylogeny-based analyses using UniFrac. Because most datasets do not meet the assumptions of normal distribution, the differences in the proportions of bacterial taxa (defined as percentage of total sequences) between mucosal and submucosal groups were determined using nonparametric Kruskal-Wallis. For comparisons involving 2 or more groups, Kruskal-Wallis and Mann-Whitney post-test were applied (non-parametric data) or ANOVA with Tukey Kramer post hoc analysis (parametric data). Comparisons between 2 groups was performed using Mann-Whitney and/or the paired Student T-test. Significance reported for any analysis was defined as p<0.05, corrected for multiple testing using ANOVA with Tukey’s HSD (honestly significant difference) post hoc analysis.

## Results

### Patient Populations

Tissues were obtained from 35 patients including 20 patients with Crohn's disease involving the terminal ileum and 15 nIBD patients as controls, as described in S1 Table. Of the 20 patients with Crohn's disease, there were 9 male and 11 females patients with a mean age of 41 (range 24–66 years of age) and mean disease duration of 16 years (range 3–34 years). Seventeen of the patients were Caucasian and 3 were African-American. Only 4 patients had received any antibiotics within 6-months of surgery while 13 patients (65%) had received anti-TNF antibody therapy. Thirteen of the 20 patients (65%) had at least one prior resection for Crohn's disease. All control tissues of the terminal ileum were obtained at time of surgery unrelated to Crohn's disease. Most control tissues were obtained from Caucasian females (11 of 15 and 10 of 15, respectively) with a mean age of 59 (range 36–88 years). There was no statistically significant correlation between the mucosal and/or submucosal microbiota and any clinical parameter in either the Crohn's disease or nIBD groups, including gender, age, ethnicity, secondary complications, concurrent disease, age of disease onset, years since disease onset, previous surgeries, previous procedures, family history of IBD, medications within the last 6-months and at time of surgery (including antibiotics, steroids, and biologics), use of pre- or probiotics, and the use of bowel cleaners at time of surgery. This lack of clinical correlation was likely the result of population size.

### Tissue Processing and DNA Extraction

The amount of surgical intestinal tissue obtained averaged 1677 mg with a range of 557 mg to 4200 mg. The average amount of tissue processed was 667 mg (+/- 139 mg) resulting in an average of 344 mg (+/- 140 mg) of submucosal tissue and an average of 240 mg (+/- 118 mg) of mucosal tissue. From these tissues, an average of 205 μg (+/- 63 μg) of DNA was obtained from the submucosal layer (~597 ng/mg) and 75 μg (+/- 37 μg) DNA obtained from the mucosal tissue (~315 ng/mg). The difference in the amount of DNA obtained from each section (almost 2x the amount of DNA from submucosal tissue as opposed to mucosal tissues) was related to the amount of human DNA within the sample (1 human genome is equivalent to 1000 bacterial genomes).

An average of 81,500 sequences were generated in each of the 70 DNA samples yielding an overall resolution down to 0.0001% of the bacterial populations (percent relative proportion of sequences) within respective tissues samples (depth of coverage). Of the 4,802,328 total sequences generated, 98.9% or 4,749,183 sequences aligned with the Kingdom Bacteria that clustered into 8545 unique sequences or OTUs with <3% divergence or operational taxonomic units (OTU’s) enabling the tentative identification of 401 bacterial genera and 698 individual bacterial species at 97% homology. The generation of 81,500 sequences per sample in this study is far greater than the 10–30,000 sequences generated in most other studies, thereby allowing a greater depth of coverage.

Of bacteria identified within intestinal tissues, 59% could be tentatively identified at the species level, 29% to the Genus level, 8% to the level of Family, 3% to the level of Order, and 2% to the level of Class. Two organisms could only be identified at the Phylum level; a single organism from the ileal mucosa of a Crohn's disease patient identified only as belonging to the Phylum *Chloroflexi* and a single organism from the ileum of a nIBD control identified as belonging to the Phylum *Negativicutes* (S1 File).

There was a significant difference (p = <0.001) in the number of sequences generated between the mucosa and submucosa in both Crohn's disease and nIBD controls, presumably resulting from the large amount of competitive human DNA present within submucosal DNA samples. The average number of sequences generated from mucosal tissues was 108,360 as opposed to 61,782 for the subjacent submucosal tissues processed side-by-side. Likewise, there was significantly more human DNA within submucosal samples than in mucosal samples (p = <0.002). The success of mucosal-submucosal separation could be predicted based on the actual sequence (fasta) counts at the species level between mucosal and submucosal tissues (Table 1). In most samples, when raw data of parallel mucosal and subjacent submucosal tissues were compared (or normalized if necessary to represent comparative numbers of sequences), inadvertent influence of microbes from mucosal tissues on the submucosal microbiota was minimal (Table 1).

**Table 1:** Differences in the number of OTU’s detected between mucosal and submucosal DNA samples suggesting that inadvertent influence of the submucosa with microbes from the mucosa was minimal.*

	Mucosa	Submucosa
*Blautia* spp.	2809 (2.72%)	6505 (6.52%)
*Campylobacter* spp.	4057 (3.92%)	45 (0.05%)
*Clostridium* spp.	3938 (3.81%)	9902 (9.93%)
*Collinsella* spp.	2951 (2.85%)	5425 (5.44%)
*Dialister* spp.	3115 (3.01%)	126 (0.13%)
*Escherichia* spp.	1474 (1.43%)	722 (0.72%)
*Prevotella* spp.	1 (<0.00%)	269 (0.27%)
*Selenomonas* spp.	3535 (3.42%)	252 (0.25%)
*Streptococcus* ssp.	274 (0.26%)	149 (0.15%)
*Tumebacillus* spp.	59 (0.06%)	4687 (4.70%)
*Parasutterella* spp.	2 (<0.00%)	93 (0.09%)
*Methylobacterium* spp.	0 (<0.00%)	381 (0.38%)
*Ruminococcus* spp.	1634 (1.58%)	2066 (2.07%)
*Veillonella* spp.	566 (0.55%)	346 (0.35%)
Total Sequences	103407	99718

*Although some contamination of submucosal tissues with mucosal microbes may occur (e.g., *Campylobacter* spp.), the high prevalence of certain genera (e.g., *Tumebacillus* spp. and others) within subjacent submucosal tissues as opposed to the parallel mucosa strongly support the notion that mucosal contamination is not a major factor contributing to the submucosal microbiota.

### Influence of rRNA operon number on relative frequency

Routine frequency analysis pipelines and bioinformatics of 16s metagenomic data based on the Illumina MiSeq and other sequencing platforms erroneously assume that all bacterial species have the same 16s ribosomal gene copy number. For example, if there are 1000 sequences and 100 of the sequences are classified as clostridia then it is assumed that clostridia represents 10% of the total population. However, bacteria of the Class *Clostridia* have an average of 6.49 copies of the16s ribosomal gene and are thus generally under-represented while other bacteria, such as bacteria of the Class *Acidobacteria*, may be over-represented.

**Fig 2:**
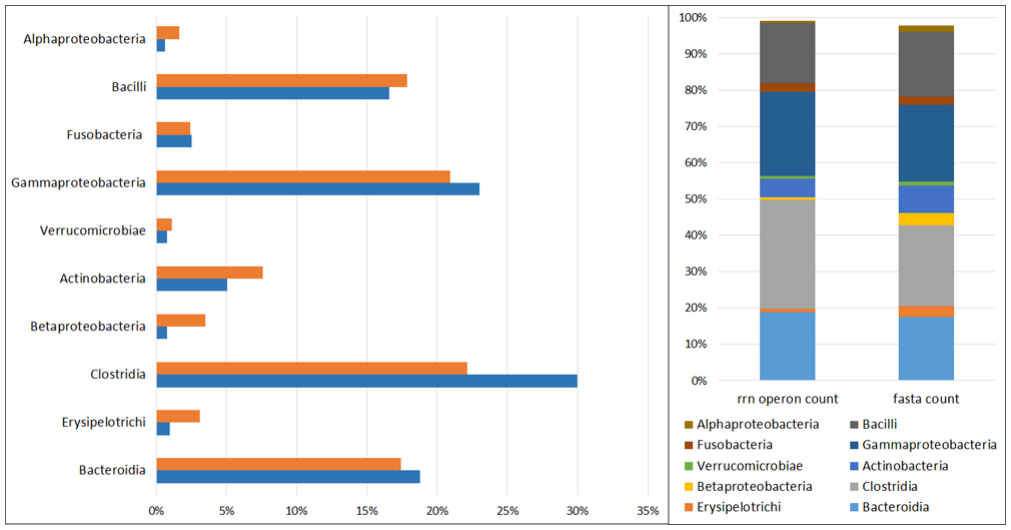
Influence and effect of rRNA gene copy number on the relative abundance of bacterial populations. Conversion of data to relative percent of population based on the number of 16s rRNA operons in each organism, thereby representing the number of bacteria or genomes, had a major effect on the composition of the bacterial communities within the mucosa and submucosa. Certain bacteria are either over or under represented when a 1:1 ratio is assumed per normal bioinformatics. Similar disparities were found at all bacterial divisions. *Legend*: Fasta Count: Relative abundance based on the assumption that each rRNA sequence alignment is equivalent to a single bacterial genome/cell as used in most bioinformatics analyses. rrn Operon Count: relative bacterial abundance based on the average number of rRNA operons in each bacterial cell (fasta count/rrn operons) thereby more accurately reflecting bacterial prevalence.

Re-calibration of bacterial frequencies within tissue populations based on the actual number of 16s rRNA operons, and hence more closely correlating with actual bacterial counts, resulted in significant changes in the relative frequencies of the microbial populations within tissues and altered the relative composition of the microbiota (Fig 2).

### Mucosal Microbiota of the Ileum in nIBD Controls

**Fig 3:**
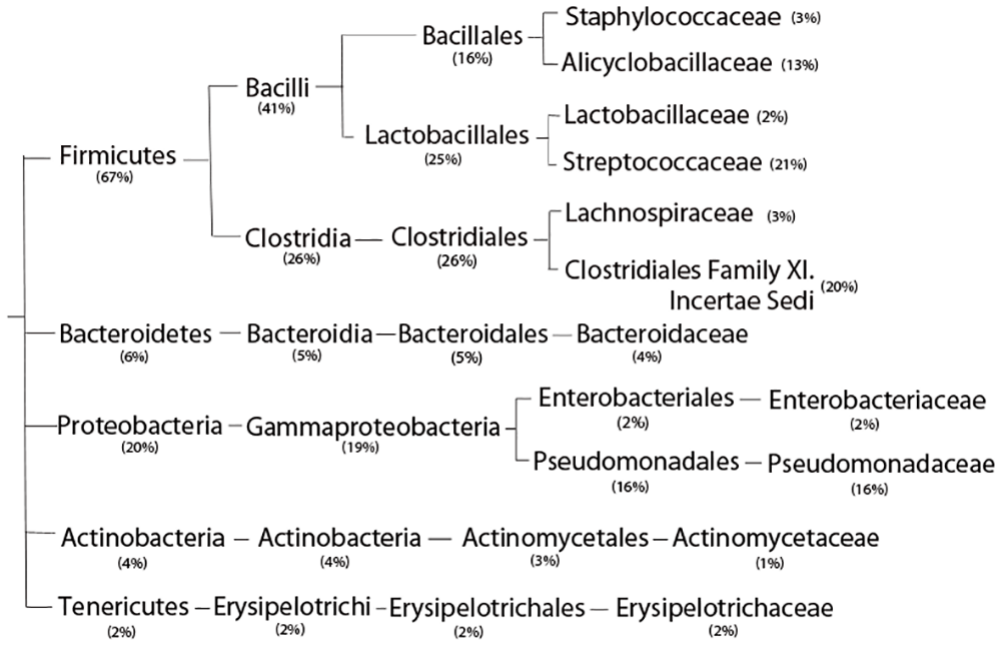
Submucosal and mucosal microbiota in nIBD controls. Major bacterial populations and average relative frequencies found in deep mucosal and submucosal ileal tissues from non-inflammatory bowel disease controls accounting for > 95% of the total microbiome. There was no statistically significant increase in bacteria within the submucosa as compared to the mucosa. Percent normalized to whole bacterial genomes based on rrn operon counts.

There was no statistically significant difference in the relative abundance of bacteria within the submucosa as compared to mucosal tissues from nIBD patients. Significant differences, as expected, were limited to increased prevalence/detection within mucosal tissues as compared to the submucosa. Predominate bacteria detected in control tissues, accounting for over 95% of the total bacterial population, normalized based on rrn copy number, are illustrated in Fig 3. There was a disproportionate amount of human DNA within submucosal samples from control ileum suggesting a very low bacterial biomass within these samples.

As expected, organisms of the Phylum *Firmicutes* (predominately anaerobic gram-positive bacteria) accounted for the majority of organisms detected representing 67% +/- 10% of the total bacterial population. Within the Phyla, organisms of the Family *Alicyclobacillaceae* (Class *Bacilli*, Order *Bacillales*), *Streptococcaceae* (Class *Bacilli*, Order *Lactobacillales*), and *Clostridiales* Family XI. Incertae Sedi (Class *Bacilli*, Order *Lactobacillales*) accounted for approximately 13%, 21%, and 20% of the microbiome respectively. Organisms of the Phylum *Proteobacteria* (gram-negative bacteria) were unexpectedly found to be a major population, with organisms of the Family *Pseudomonadaceae* (Class *Gammaproteobacteria*, Order *Pseudomonadales*) accounting for 16% of that division. These findings suggest that the control tissues used in the present study may not be completely normal and may have active inflammation suggested by higher than expected prevalence of Proteobacteria [17,18]. Other major bacterial populations included organisms of the Phylum *Bacteroidetes* (6%), *Actinobacteria* (4%) and *Tenericutes* (2%). (Fig 3).

In addition to the major bacterial groups identified above and in Fig 3, the minor bacterial populations which accounted for less than 5% of the total population, were represented by 133 different bacterial genera and 473 tentative species in the control ileum group. Most of these genera and species were found at low relative abundance randomly in certain individuals not correlated with any clinical parameters and likely represent bacteria exclusive to the individual’s social habits, including diet.

### Mucosal Microbiota of the Ileum in Crohn's disease as compared to nIBD Controls

**Fig 4:**
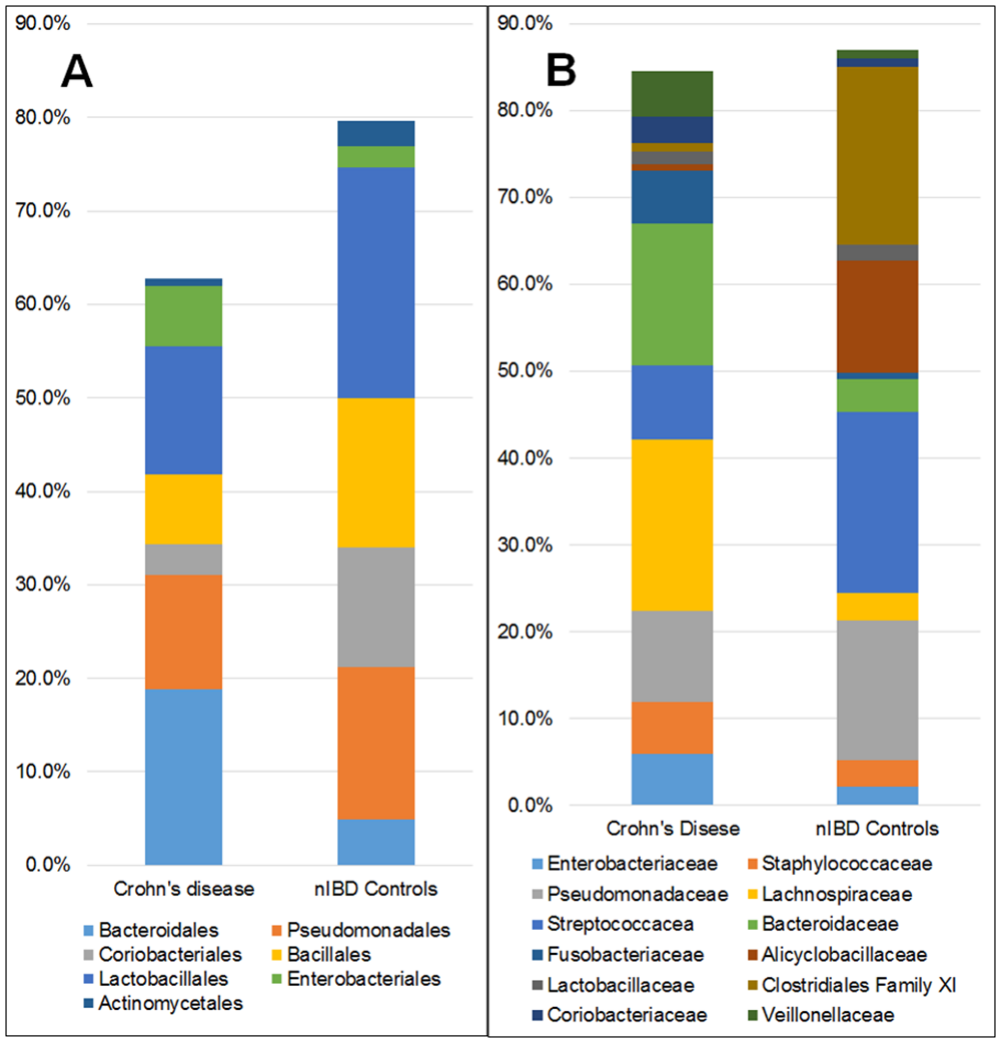
Differences in the relative abundance of bacterial Orders (A) and Families (B) in the mucosa of patients with ileal Crohn's disease compared to the ileal mucosa in nIBD controls. Except for an increase in bacteria of the Order *Bacteroidales* and *Enterobacteriales*, mucosa from ileal Crohn's disease patients had a reduced abundance of most other bacteria. There were major differences in most bacterial Families between Crohn's disease and controls. Illustrated are only bacteria comprising > 1% of the total bacterial population and present in >50% of patients.

The relative abundance of bacterial populations in the mucosa of patients with Crohn's disease were significantly different from that found in controls, particularly farther down the phylogenic tree (Fig 4). At the Phylum level, only *Bacteroidetes* were significantly increased (p = 0.002) and organisms within this Phylum remained a statistically significant population down to the family level. These findings only loosely correlated with those reported in other studies. Most studies have reported decreases in organisms of the Phylum *Firmicutes* with accompanying increases in *Proteobacteria* and *Bacteroidetes* in Crohn's disease [2,19]. Although Firmicutes were reduced and *Proteobacteria* were increased in Crohn's disease mucosa, they were not significantly different from nIBD control mucosa (p >0.05).

Significant differences between diseased mucosal tissues from Crohn's disease and control mucosa included increases in members of the Order *Bacteroidales* (p = 0.003) and *Coriobacteriales* (p = 0.03) and members of the Family *Lachnospiraceae* (p = 0.001) and *Bacteroidaceae* (p = 0.005) in Crohn's disease mucosa. Trends toward statistically significant increases in Crohn's disease were also observed for Order *Enterobacteriales* (p = 0.06) and Family *Enterobacteriaceae* (p = 0.056). Significant decreases of bacteria in the mucosal tissues from Crohn's disease included members of the Family *Alicyclobacillaceae* (p = 0.01) and *Clostridiales* Family XI. Incertae Sedi (p = 0.01) (Fig 4).

In addition to differences in the relative abundance and frequency of certain bacterial divisions, several bacterial genera/species appeared to be present only in Crohn's disease and not in any control tissues. For instance, Organisms of the Family *Desulfovibrionaceae* were detected only in the Crohn's disease group and not in any controls.

### Submucosal microbiota as compared to the corresponding mucosa within the diseased Ileum

**Fig 5:**
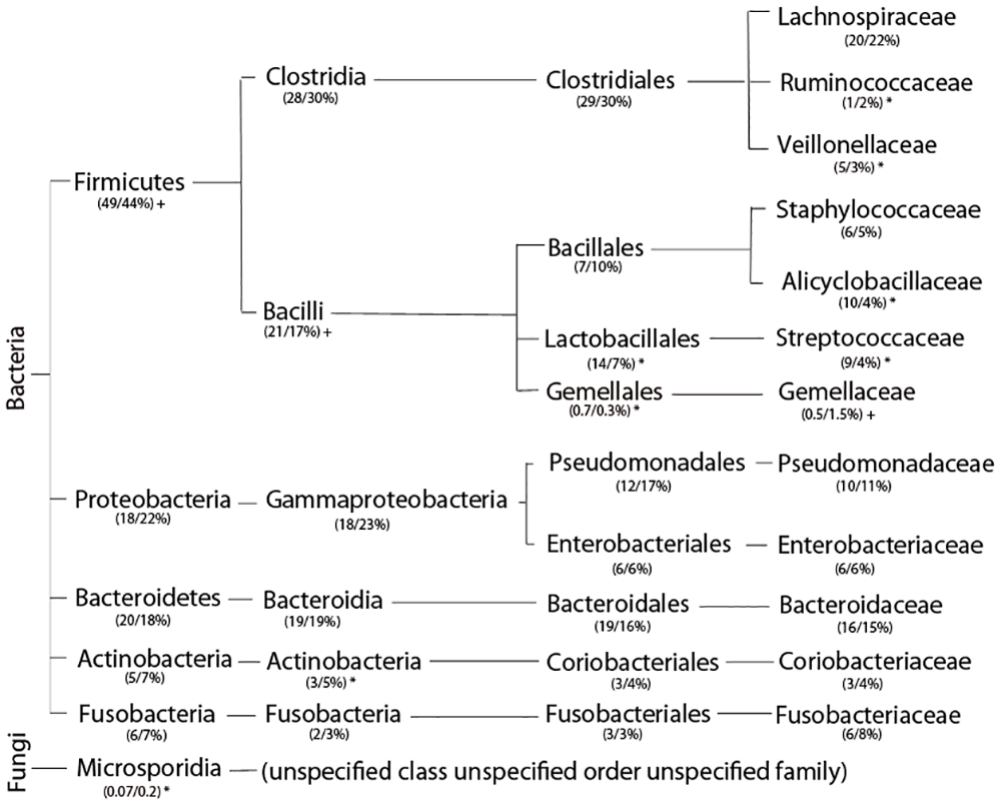
Bacterial populations of the diseased submucosa and mucosa at the center of the diseased tissues in Crohn's disease. The values in parentheses represent the average relative frequency in mucosa/submucosa. An asterisk (*) denotes that the difference between the mucosa and submucosa was statistically significant (p = <0.05) while a plus (+) represents a trend toward statistical significance (p = >0.05 but <0.065).

The predominate organisms that accounted for 95% of the relative bacterial populations of the diseased mucosa and submucosa are illustrated in Fig 5.

There was little difference between the microbial populations of the mucosa and submucosa at the Phylum level. The only statistically significant difference noted at the Phylum level was an increase in the abundance of *Microsporidia* (Kingdom *Fungi*) in the submucosa as compared to the mucosa (p = 0.04) which remained significantly increased within the submucosa at the Class, Order, and Family level as *Microsporidia* unspecified Class unspecified Order unspecified Family (p = 0.04, 0.03, 0.04, respectively).

**Fig 6:**
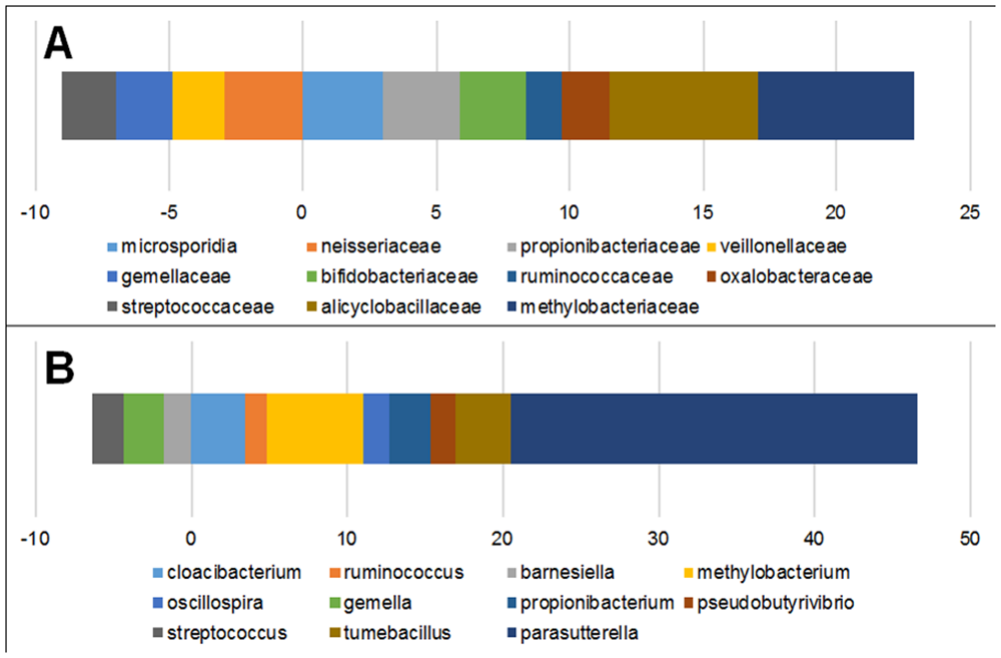
Certain bacterial families have a greater tendency to transgress mucosal barriers and colonize submucosal tissues. Relative fold increase/decrease in the abundance of bacterial Families (A) and Genera (B) within the subjacent ileal submucosa as compared to the parallel superjacent mucosa from tissues in the center of the disease lesion suggesting the ability of certain bacteria to invade and colonize the diseased submucosal tissues. Four families and 8 Genera had a predilection to penetrate mucosal barriers and colonize the submucosa while 4 Families and 3 Genera had a diminished capacity to invade submucosal tissues. *Parasutterella* spp. (dark blue, panel B) had a particular predilection to penetrate mucosal barriers. *Legend*: Bacteria present in less than 50% of patients are excluded. All increases/decreases were statistically significant (p = <0.05) except as noted with an asterisk (*) in which p = >0.05 but <0.065 suggesting a trend toward statistical significance.

Organisms of the Class *Actinobacteria* and *Sphingobacteria* were increased while organisms of the Order *Gemellales* (p = 0.04) and *Lactobacillales* (p = 0.01) were reduced in the submucosa of diseased tissues as compared to the paired mucosa. Major differences between the submucosa and mucosa did not become clearly evident until the Family level where *Propionibacteriaceae* was increased 3-times and *Alicyclobacillaceae* increased over 5-times in the submucosa as compared to the mucosa (p = 0.04). Additionally, *Ruminococcaceae* (1.5x increase; p = 0.04), *Oxalobacteraceae* (2x increase; p = 0.02) and *Microsporidia* (3x increase; p = 0.05) were increased in the submucosa while *Veillonellaceae* (2x decrease; p = 0.009), *Gemellaceae* (2x decrease; p = 0.02), and *Streptococcaceae* (2x decrease; p = 0.02) were reduced in the submucosa as compared to the mucosa (Fig 6).

At the genus level, a variety of organisms of the Phylum *Firmicutes* were increased in the submucosa as compared to the paired mucosa including *Ruminococcus* spp. (1.4 fold increase; p = 0.03), *Oscillospira* spp. (1.6 fold increase; p = 0.01), *Pseudobutyrivibrio* spp. (1.6 fold increase; p = 0.03), and *Tumebacillus* spp. (3.6 fold increase; p = 0.04). In addition, *Propionibacterium* spp. (2.7 fold increase; p = 001) and *Cloacibacterium* spp. (3.5 fold increase; p = 0.05) were increased in the submucosa as well as large increases in *Proteobacteria* including *Parasutterella* spp. (26.1 fold increase; p = 0.04) and *Methylobacterium* spp. (6.1 fold increase; p = 0.02) (Fig 6B). Bacteria belonging to the genera *Barnesiella* (1.8 fold decrease; p = 0.04), *Gemella* (2.5 fold decrease; p = 0.03), and *Streptococcus* (2.0 fold decrease; p = 0.02) were decreased in the submucosa.

The large increase in *Parasutterella* spp. within the submucosa (26 fold increase as compared to the mucosa) (Fig 6B) undoubtedly accounted for much of the increase in *Proteobacteria* within submucosal tissues. Operational taxonomic units corresponding to *Parasutterella* spp. did not match any known species of the genus. Organisms belonging to the genera *Salmonella*, *Shigella*, *Helicobacter*, *Listeria*, *Yersinia*, *or Vibrio* were not detected in any Crohn's disease patients or controls. *Campylobacter* spp., predominately *C*. *gracilis* and *C*. *concisus*, were detected within the mucosa in 65% of Crohn's disease patients. Although *Campylobacter* spp. were not detected in any ileal controls used in the present study, they were detected in (40%) of nIBD colon samples not included in the present study.

Although similarities existed between the mucosal and submucosal tissues, the bacterial populations of the deep submucosa were only loosely reflected in the microbiota of the mucosa. Most of the bacteria identified as increased or decreased within the mucosal tissues showed no significant difference with the microbial populations within the submucosa and some bacteria, such as *Gemellaceae*, *Veillonellaceae*, and *Neisseriaceae* that were increased within the mucosal layer were actually decreased in the submucosa (Fig 6A) suggesting that these bacteria are unable, or have a diminished capacity, to penetrate the mucosal barrier and colonize the submucosal spaces of the ileum. In contrast, bacteria of the Family *Alicyclobacillaceae* (Phylum *Firmicutes*, Class *Bacilli*, Order *Bacillales*), which were decreased in the mucosa and represented only 0.65% of the bacterial population, increased over 25-fold in the submucosa to reach a relative abundance of 4.5% of the submucosal microbiota.

In addition to elevated abundance of bacteria in the Families *Alicyclobacillaceae*, *Oxalobacteraceae* (p = 0.02), *Ruminococcaceae* (p = 0.04), and *Propionibacteriaceae* (0.04), unclassified fungi of the Phylum *Microsporidia* (unclassified Class unclassified Order unclassified Family), were almost 3 times greater in the submucosa as compared to the mucosa (Fig 6A). Although *Microsporidia* was detected within the mucosa in almost all cases including nIBD controls, it was only in the Crohn's disease group that there was a statistically significant increase within the submucosa as compared to the mucosa (p = 0.04). These findings may support microspordiosis as a compounding factor in Crohn's disease [20].

### The submucosal microbiota in Crohn's disease as compared to nIBD Controls

As the mucosal microbiota was significantly different between nIBD controls and Crohn's disease (Fig 4), there were major differences in the submucosal microbiota between Crohn's disease and nIBD controls at all taxonomic levels.

**Fig 7:**
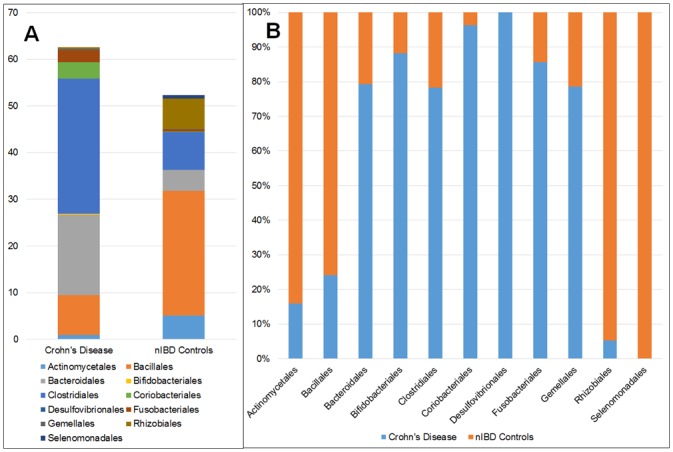
Comparison of the relative abundance of bacterial Orders within the submucosa of the diseased ileum in Crohn's disease as compared to the ileal submucosa in nIBD controls. Various Orders, such as *Selenomonadales* and *Rhizobiales*, are absent or poorly represented with the mucosa of Crohn's disease tissue while other orders such as *Desulfovibrionales* and *Coriobacteriales* are absent or poorly represented in nIBD controls. Panel A: Relative abundance within submucosal tissues of each bacterial Order in each disease group. Panel B: Relative comparison and contribution of each Order to the total. *Legend*: Bacteria present in less than 50% of patients are excluded. All data presented are statistically significant (p = <0.05).

Most notably, at the Phylum level, *Tenericutes* were 60-fold higher (p = 0.001) and *Fusobacteria* were 6-fold higher (p = 0.01) within Crohn's disease submucosal tissues as compared to nIBD controls. At the taxonomic level of Class, there were significant differences in 7 of the 34 classes detected including increases within the submucosa of *Bacteroidia* (4 fold increase; p = 0.009), *Clostridia* (3.5 fold increase; p = 0.007), *Fusobacteria* (5.6 fold increase; p = 0.03) as compared to nIBD controls. In addition, *Coriobacteria* were detected in 17 of 20 (85%) submucosal tissues from Crohn's disease but not in the submucosa of any nIBD control (p = 0.004). Conversely, *Negativicutes* were detected in the submucosa of 11 of 15 nIBD controls (79%) but not in the submucosa of a single Crohn's disease patient. Similar changes were noted at the Order taxonomic level with major differences between the submucosal microbiota in ileal Crohn's disease and in nIBD controls (Fig 7) including a 25-fold increase in bacteria of the Order *Coriobacteriales* (p = 0.001) and a 6–7 fold increase in bacteria of the order *Bifidobacteriales* (p = 0.03) and *Fusobacteriales* (p = 0.02) within the submucosa of Crohn's disease as compared to nIBD controls. As was noted at the Class level, organisms of the Order *Selenomonadales* and *Rhizobiales* were absent or near absent in the submucosal tissue of Crohn's disease as opposed to nIBD controls (p = 0.003 and p = 0.002, respectively) and, conversely, bacteria of the order *Coriobacteriales* and *Desulfovibrionales* present in nIBD controls where absent or near absent within the submucosal tissues in Crohn's disease (p = 0.001 and p = 0.02, respectively) (Fig 7).

**Fig 8:**
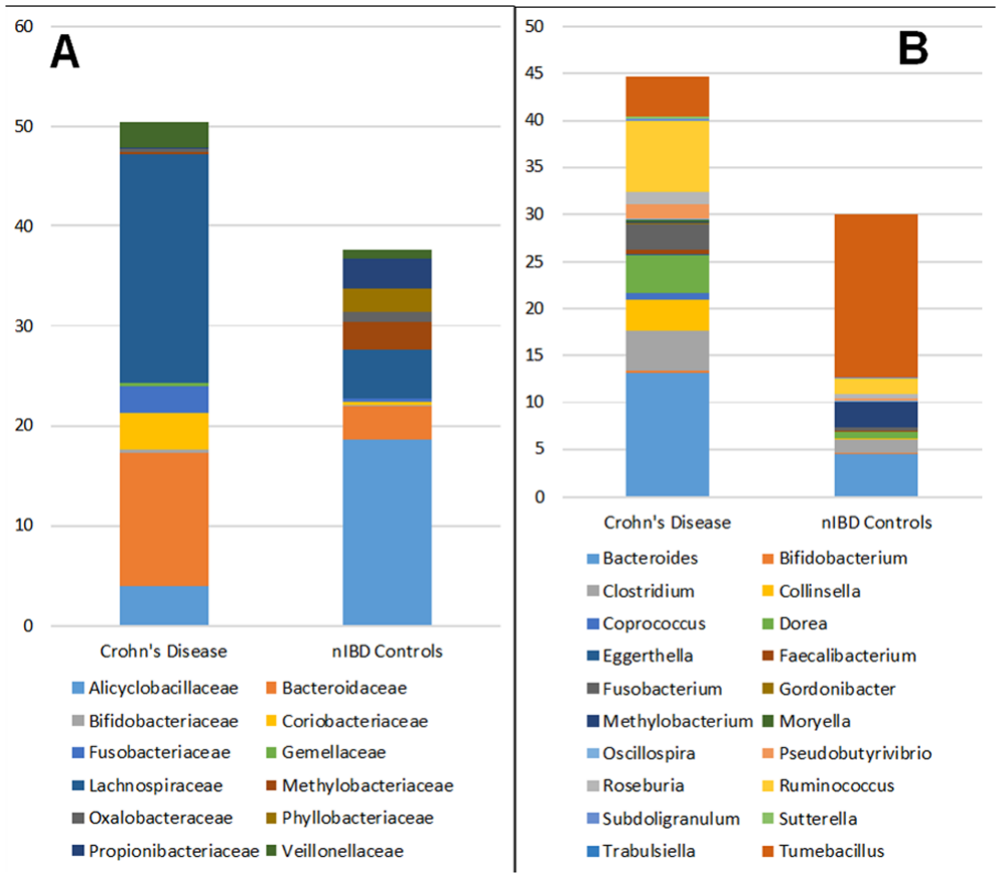
Comparison of the relative abundance of bacterial Families (A) and Genera (B) within the submucosa of the diseased ileum in Crohn's disease as compared to the ileal submucosa in nIBD controls. There were major changes in 12 families and 20 genera. *Legend*: Bacteria present in less than 50% of patients are excluded. All data presented are statistically significant (p = <0.05).

As found within higher taxonomic levels, there were statistically significant differences in bacterial populations and their prevalence at the Family and Genus levels with increased prevalence within the submucosa of Crohn's disease as compared to nIBD controls in 12 Families and 34 Genera (Fig 8). Organisms of the Genera *Gordonibacter* spp. (Phylum *Actinobacteria*) and *Trabulsiella* spp. (Phylum *Proteobacteria*) were detected within the submucosa in 12 of 20 (60%; p = 0.02) and 8 of 20 (40%; p = 0.02) patients with Crohn's disease, respectively, but not in the submucosa of a single nIBD control.

## Discussion

This is the first study to examine the microbial populations within the diseased and inflamed submucosal tissues of patients with Crohn's disease of the ileum and to compare microbial communities found deep within the submucosal tissues with those present in and on the superjacent mucosal surface.

We hypothesized that, since Crohn's disease was a transmural disease affecting all layers of the intestinal tract, the examination of submucosal tissues directly associated with the inflammation and disease process may provide insight into relevant bacterial populations capable of transgressing intestinal barriers and may more accurately reflect causes and agents of the disease. Unlike superficial snip biopsies and downstream fecal material which may face limitations in identifying bacterial populations directly involved in disease initiation and progression, we hypothesized that bacteria present deep within the diseased submucosal would be less affected by medical treatments, bowel cleaning, and other temporary modulators of the intestinal microbiota.

Thus, the submucosal microbiota at the site of primary disease represents a logical area of investigation to determine bacterial populations within unique biological niches in direct contact with the hosts’ immune system and directly associated with the inflammatory lesion. This is an area of the intestinal tract that had not previously been examined in Crohn's disease or in any other diseased or healthy population. The proposition that a submucosal microbiome exists in health and disease, distinct from the luminal and mucosal microbiome, is corroborated by the recent discovery and recognition of a broad tissue microbiota [21]. Our data demonstrates the existence of a distinct submucosal microbiota and ecosystem and that the methodologies employed confirm the existence of a submucosal microbiota that is not well reflected in the mucosa and/or downstream fecal material.

Contamination is always a concern when dealing with prokaryotic life because of their abundance and widespread distribution. Studies involving the mucosal microbiota [22–24] cannot rule out contamination of the mucosal layer with bacteria from feces and/or the mucus lining, or separate microbiota within the mucosa as opposed to those simply adherent to the mucosal surface or trapped within the mucus layers. Similarly, we cannot rule out contamination of the submucosa by bacteria arising from mucosal surfaces which presumably have a high bacterial load. Although we cannot completely rule out bacterial contamination of the submucosa by bacteria arising from the mucosa, evidence suggests that such contamination, if any, was minimal and did not adversely affect the results.

Our samples represented parallel superjacent mucosa and subjacent submucosal samples that were carefully dissected and separated. If we assume that the submucosa is a sterile environment, contamination of the submucosa by bacteria arising from the mucosa would result in equal relative prevalence of the bacterial populations detected. This is essentially what we found in comparing the mucosa and submucosa of nIBD controls, i.e, there was no statistically significant difference in bacterial prevalence between the submucosa and mucosa. In contrast, there were significant differences in the bacterial prevalence within the subjacent submucosa in Crohn's disease which, mathematically, could not occur in the absence of a previously existing bacterial population within the subjacent submucosa. In other words, a population of bacteria at different prevalence must have been present within submucosal tissues to alter bacterial populations if contamination, which would be uniform, were to occur. Thus, mathematically, the differences in bacterial prevalence observed between superjacent mucosa and subjacent submucosa in Crohn's disease cannot be accounted for by contamination (Table 1).

Thus, the differences observed between subjacent submucosa and superjacent mucosa cannot be explained by contamination and must reflect true differences in the ability of certain bacteria to penetrate and survive within the intestinal submucosal tissues of patients with Crohn's disease.

A variety of investigations looking at the microbiota in downstream fecal material and in biopsies have clearly established that the microbiota in Crohn's disease is dysbiotic [22]. Although there is diversity in data reported, particularly at lower phylogenetic levels, most studies report decreased diversity and prevalence in the Phyla *Firmicutes* accompanied by an increase in bacteria of the Phyla *Bacteroidetes*, *Proteobacteria*, and *Actinobacteria* as compared to control populations. The most comprehensive studies have been conducted in new-onset treatment-naïve pediatric patients which suggested increases in bacteria of the Families *Enterobacteriaceae*, *Pasteurellaceae*, *Veillonellaceae*, *Neisseriaceae*, *Gemellaceae*, and *Fusobacteriaceae* and decreases in bacteria of the Orders *Erysipelotrichales*, *Bacteroidales*, *Lachnospiraceae*, *Bifidobacteriaceae*, and *Clostridiales* (Table 2) [23,24]. An increase in bacteria of the Family *Enterobacteriaceae* was observed only in the presence of inflammation, supporting the notion that *Proteobacteria*, particularly *Enterobacteriaceae*, is a conserved ecological pattern generally associated with inflammation [17,18] and not disease specific.

**Table 2:** Comparison of changes in the mucosal and submucosal microbiome in Crohn's disease versus non-inflammatory bowel disease controls as reported in the present study and in treatment-naïve new-onset patients.

	Treatment-naïve new-onset		Advanced disease	
			Mucosa	Submucosa*
	Gevers *et al* [23]	Haberman *et al* [24]	Present Study	
Enterobacteriaceae	Increased	Increased	Increased	NS^1^
Veillonellaceae	Increased	Increased	Increased	Increased
Gemellaceae	Increased	Increased	Increased	Increased
Erysipelotrichales	Decreased	Decreased	NS	NS
Bacteroidales	Decreased	Decreased	NS	Increased
Clostridiales	Decreased	Decreased	NS	Increased
Erysipelotrichaceae	Decreased	Decreased	NS	NS
Pasteurellaceae	Increased	Increased	NS	NS
Fusobacteriaceae	Increased	Increased	NS	Increased
Neisseriaceae	Increased	Increased	NS^2^	NS^2^
Lachnospiraceae	Decreased	Decreased	Increased	Increased
Coriobacteriaceae	NS	NR^3^	Increased	Increased
Bifidobacteriaceae	Decreased	Decreased	NS	Increased
Actinomycetales	NR	NR	Decreased	Decreased
Bacillales	NR	NR	Decreased	Decreased
Desulfovibrionales	NR	NR	NS	Increased^4^
Bacteroidaceae	NR	NS	Increased	Increased

* Diseased submucosa vs. nIBD submucosa;

^1^ NS, no significant difference;

^2^ Present in less than 50% of both Crohn's disease and nIBD patients;

^3^ NR, not reported;

^4^ Not detected in submucosa of nIBD controls.

Our results, both within mucosal and submucosal tissues, differed substantially from that reported in biopsy specimens from new-onset treatment-naïve pediatric patients. Within the mucosa, there was agreement only on the increased prevalence of *Enterobacteriaceae*, *Veillonellaceae*, and *Gemellaceae* and, in the submucosa, only with *Veillonellaceae*, and *Gemellaceae* (Table 2). Major differences within the mucosa also included increased abundance of bacteria of the Families *Coriobacteriaceae* (Phylum *Actinobacteria*, Class *Actinobacteria*, Order *Coriobacteriales*), *Lachnospiraceae* (Phylum *Firmicutes*, Class *Clostridia*, Order *Clostridiales*) and *Bacteroidaceae* (Phylum *Bacteroidetes*, Class *Bacteroidia*, Order *Bacteroidales*) and decreased prevalence of *Actinomycetales* (Phylum *Actinobacteria*, Class *Actinobacteria*) and Bacillales (Phylum *Firmicutes*, Class *Bacilli*) not previously reported as significantly different within the mucosal surfaces in Crohn's disease (Table 2).

The differences in the intestinal mucosal microbiota we observed as compared to those previously reported by other investigators likely resulted from a variety of compounding factors, including, but not limited to, advanced disease compounded with the effects of long-term chemotherapeutic manipulations. Foremost, the microbiota present within submucosal tissues have not previously been examined (Fig 1) such that differences may not be relatable. Other factors which may account for these differences include differences in control populations, normalization of frequency data based on rRNA operon numbers which is known to affect prevalence [14], DNA extraction methods, rRNA universal primers used, sequencing methodologies, depth of coverage and OTU’s generated (median sequence depth), and databases used for alignment and bioinformatics [25]. Most studies report median sequence depths of 10,000–30,000 sequences per sample [23,24] while in this study we generated an average of 81,500 sequences per sample. Differences in sequence depths, in addition to the deeper submucosal tissues examined, would undoubtedly affect and influence the relative abundance of bacterial populations within the intestinal microbiota.

Our 16s rRNA data was recalibrated to reflect bacterial genomes rather than simply reflecting rRNA target numbers which is not an accurate representation of bacterial counts. While recalculation did not make a difference when comparing identical bacteria, it made a significant difference when calculating relative percent prevalence. For example, organisms of the Family *Coriobacteriaceae* are reported as representing 3.8% of the population, but when the data is normalized based on rRNA gene copies, the true prevalence is 8.2% (p = 0.003). These findings support the suggestion that incorporating rRNA gene copy number in bacterial community analyses improves estimates of microbial diversity and abundance [15, 16].

Since Crohn's disease is a recurring chronic disease (65% of our patient population had prior surgical removal of disease), if the etiology of Crohn's disease has a bacterial origin, then the bacterial agent(s) responsible would likely still be present in advanced as well as new-onset treatment-naïve patients. Alternately, if the microbiota composition of advanced disease is considered not to be significant due to long-term therapeutic interventions [23,24], then it must also be concluded that the altered intestinal microbiota and dysbiosis is secondary to the disease process. Accordingly, the examination of submucosal tissues in advanced disease is relevant to understanding the etiopathogenesis of disease and identification of causative agents and factors.

The purpose of the present study was not to compare the mucosal microbiota in Crohn's disease to normal ileal mucosa as in previous studies [22], but to compare the microbiota of the mucosa to the corresponding paired subjacent submucosal tissues. Although the submucosal microbiota generally reflected the dominant bacterial divisions of the mucosal tissues and visa-versa, the mucosal microbiota did not accurately predict or reflect those bacterial families or genera that were increased in relative prevalence within submucosal tissues.

Most of the organisms significantly elevated within the submucosa belonged to the Phyla *Firmicutes* (Gram-positive facultative or obligate anaerobes) and *Proteobacteria* (Gram-negative facultative anaerobes), with smaller representation by bacteria of the Phylum *Bacteroidetes* and *Actinobacteria*. We identified 4 Families of bacteria that were significantly increased and 2 Families of bacteria with a trend toward statistical significance within the diseased submucosal tissues as compared to the mucosa suggesting invasion and colonization within the tissues and not merely adherent to the mucosal surface (Fig 6). These Families were reflected in 8 Genera that were increased within submucosal tissues including bacteria of the Order *Desulfovibrionales* (Phyla *Proteobacteria*, Class *Deltaproteobacteria*), and all lower classifications, which were exclusively found within the submucosa of the Crohn's disease group. Although detected within the mucosa of 70% (14 of 20) of Crohn's disease and in only 27% (4 of 15) nIBD controls, there was no significance difference in detection or prevalence within mucosal tissues. This organism was present in 55% (11 of 20) submucosal samples from patients with Crohn's disease at an average prevalence of 0.01% and absent in all 15 submucosal samples from nIBD controls. There was a significant difference at the Order (p = 0.02) and Family (p = 0.04) phylogenic levels, and the organism was most closely related to *Bilophia* spp. [26] (p = 0.04). Although little is known about the Genus *Bilophia*, *Bilophia wadsworthia* is able to adhere to human intestinal cells and has been associated with a variety of human and animal infections [26–28].

It is worthy to note that although organisms of the Family *Enterobacteriaceae* were increased within mucosal tissues as consistently reported in other studies, they were not increased within the submucosa suggesting that these microbes do not have a predilection to invade deep submucosal tissues. These findings raise questions related to the implication of adherence-invasion genes associated with this group of bacteria [7] and the increased prevalence of adherent-invasive *E*. *coli* (AIEC) in Crohn's disease [29]. Further studies will be required to determine if organisms of the Phylum *Proteobacteria* are specifically relevant to Crohn's disease or simply reflect a conserved ecological pattern related to inflammation [17,18].

Our data further supports the notion that it may not be appropriate to assume that fecal microbiota and mucosa-adherent bacterial populations are realistic surrogates for the entire gut microflora and that distinct niches and ecosystems exist within specific anatomical sites within the intestines. As shown herein (Table 2), tissue associated, surface-adherent, and luminal microbial populations are distinct and likely fulfill different roles within the ecosystem.

Larger patient populations will be required to determine if the findings herein reported can be validated and correlated with clinical features to determine if the identification of bacterial populations associated with the inflammatory process within the submucosa, and likely contributing to the disease process, will lead to the discovery of microbial populations that will contribute to diagnostic procedures and targeted therapy to return the dysbiotic intestinal microbiota to eubiosis.

## Supporting Information

S1 Table Characteristics of Crohn's disease patient population. (DOCX)

S1 File Level of identification of all bacteria within mucosal and submucosal tissues. (XLSX)
