# Supplementary material for: Microbial Population Differentials between Mucosal and Submucosal Intestinal Tissues in Advanced Crohn's Disease of the Ileum
Source: PLoS One. 2015 Jul 29;10(7):e0134382. doi: 10.1371/journal.pone.0134382 (PMC4519195; doi:10.1371/journal.pone.0134382)
Supplement: S1 Table — (DOCX) [file pone.0134382.s001.docx]

| **S1 Table. Characteristics of Crohn's disease patient population**. From each patient, tissues were obtained at time of surgery from full thickness intestinal sections representing the center of the diseased area. ^1^ | | | | | | | | |
| --- | --- | --- | --- | --- | --- | --- | --- | --- |
| **Patient** | **Age** | **Sex** | **Race** | **Years of Disease** | **Current Medications**  **(at time of surgery)** | **Medications**  **(last 6-months)** | **Disease site** | **Prior surgery** |
| CD001 | 47 | M | Caucasian | 20 | 6mp, prednisolone | 6mp, prednisolone | Ileum | YES |
| CD002 | 49 | F | Caucasian | 24 | ASA, Budesonide | ASA, Budesonide | ileocolonic | YES |
| CD003 | 36 | M | Caucasian | 25 | Imuran (1 wk prior) | Remicade | T. Ileum | YES |
| CD004 | 35 | M | Caucasian | 13 | Ciprofloxacin | Ciprofloxacin | ileocolonic | NO |
| CD005 | 32 | F | Caucasian | 5 | Imuran (5 days prior) | Remicade (8 Wk prior) | T. Ileum | NO |
| CD007 | 38 | M | Caucasian | 19 | none | none | small bowel | YES |
| CD009 | 36 | F | Caucasian | 14 | prednisolone | prednisolone | T. ileum | NO |
| CD011 | 43 | M | African-Am | 15 | prednisolone | prednisolone | ileocolonic | NO |
| CD013 | 24 | M | Caucasian | 6 | ASA | ASA | T. Ileum | YES |
| CD012 | 52 | F | Caucasian | 14 | Budesonide | Budesonide | T. Ileum | YES |
| CD014 | 49 | M | Caucasian | 2 | Humira | Humira | T. Ileum | NO |
| CD015 | 66 | F | Caucasian | 25 | prednisolone | prednisolone | T. Ileum | YES |
| CD016 | 43 | F | African-Am | 3 | Humira, ASA, prednisolone, Imuran | none | T. Ileum | YES |
| CD017 | 55 | M | Caucasian | 34 | none | methotrexate, Budesonide | T. Ileum | YES |
| CD018 | 45 | M | Caucasian | 22 | Humira | Humira | T. Ileum | YES |
| CD019 | 30 | F | Caucasian | 1 | ASA, Humira (4 wk prior), rifaximin | ASA, Humira, rifaximin | T. Ileum | NO |
| CD021 | 24 | F | Caucasian | 11 | Humira, flagyl, ASA | Humira, flagyl, ASA | T. Ileum | NO |
| CD030 | 50 | F | Caucasian | 14 | Budesonide | Budesonide | T. Ileum | YES |
| CD034 | 31 | F | Caucasian | 17 | hydrocortizone, Doxycycline, flagyl, piperacillin, tazobactam | Remicade | ileum | YES |
| CD036 | 51 | F | African-Am | 27 | 6mp | prednisolone | T. Ileum | YES |
|  |  |  |  |  |  |  |  |  |
|  | | | | | | | Tissue. Reason for surgery | |
| nIBD026 | 69 | F | Caucasian | <1 | Pepcid | Pepcid | T. ileum. Ileostomy takedown post radiation enteritis | |
| nIBD027 | 62 | F | Caucasian | <1 | Fluoxetine(Prozac), Diovan, Prednisolone | Hydrochlorothiazide  Olanzapine, Fluoxetine | T. ileum. Ileostomy reversal post C. diff complications | |
| nIBD029 | 69 | F | Caucasian | 9 | Synthroid, metformin, **Nortriptyline, Macrodantin, Albuterol, metoprolol, Zocor, ASA, Prozac, estradiol** | Same as current | T. ileum. Ileostomy reversal post colonoscopy perforation | |
| nIBD032 | 81 | F | Caucasian | 1 | Lovenox, synthroid, diovan, Toprol, Celebrex, lipitor | Same as current | T. ileum. Ileostomy reversal post C. diff complications. | |
| nIBD046 | 32 | F | African-Am | <1 | Bactrim, Clindamycin, Diphenoxylate, Prilosec, Flexeril, Phenergan, citalopram, benadryl | Same as current | T. ileum. Ileostomy reversal post trauma | |
| nIBD047 | 52 | F | Caucasian | <1 | ranitidine, claritin | ranitidine | T. ileum. Ileostomy reversal post cancer | |
| nIBD052 | 60 | F | Caucasian | <1 | Ciprofloxacin | Same as current | T. ileum. Stenosis of ileostomy post ulcerative colitis | |
| nIBD055 | 36 | F | Caucasian | 4 | Norco, Neurontin | Same as current | T. ileum. Ileostomy reversal post trauma | |
| nIBD056 | 64 | M | Caucasian | >1 | Ciprofloxacin, Ambien, Dexilant, lomotil, Norco, synthroid, xanax | Flagyl | T. ileum. Ileostomy reversal post cancer | |
| nIBD058 | 44 | F | Caucasian | 1 | Linzess, ondansetron, hydrocodone, vivelle-dot | Same as current | T. ileum. Ileostomy reversal post cancer | |
| nIBD059 | 52 | F | Caucasian | <1 | Flomax, pantoprazole, Zofran, phenazopyridine | Ampicillin | T. ileum. Ileostomy reversal post diverticulitis | |
| nIBDTT02 | 60 | M | Hispanic | <1 | None | None | T. ileum. Colon cancer | |
| nIBDTT03 | 60 | M | Hispanic | <1 | None | None | T. ileum. Colon cancer | |
| nIBDTT04 | 56 | M | Hispanic | <1 | None | None | T. ileum Appendix cancer | |
| nIBDTT05 | 88 | F | Hispanic | <1 | None | None | T. ileum. Colon cancer | |
| CD, Crohn’s Disease; nIBD, non-inflammatory bowel disease control; NK, not known; Am, American; 6mp, 6-mercaptopurine; ASA, aminosalicylic acid; T. ileum, terminal ileum, C. diff, *Clostridium difficile* | | | | | | | | |
